# Life on a leaf: the epiphyte to pathogen continuum and interplay in the phyllosphere

**DOI:** 10.1186/s12915-024-01967-1

**Published:** 2024-08-07

**Authors:** Graham Thomas, William T. Kay, Helen N. Fones

**Affiliations:** 1 Biosciences, University of Exeter, Exeter, UK; 2 Department of Plant Sciences, University of Oxford, Oxford, UK

**Keywords:** Leaf, Plant pathogen, Phyllosphere, Epiphytic, Microbiome

## Abstract

Epiphytic microbes are those that live for some or all of their life cycle on the surface of plant leaves. Leaf surfaces are a topologically complex, physicochemically heterogeneous habitat that is home to extensive, mixed communities of resident and transient inhabitants from all three domains of life. In this review, we discuss the origins of leaf surface microbes and how different biotic and abiotic factors shape their communities. We discuss the leaf surface as a habitat and microbial adaptations which allow some species to thrive there, with particular emphasis on microbes that occupy the continuum between epiphytic specialists and phytopathogens, groups which have considerable overlap in terms of adapting to the leaf surface and between which a single virulence determinant can move a microbial strain. Finally, we discuss the recent findings that the wheat pathogenic fungus *Zymoseptoria tritici* spends a considerable amount of time on the leaf surface, and ask what insights other epiphytic organisms might provide into this pathogen, as well as how *Z. tritici* might serve as a model system for investigating plant–microbe-microbe interactions on the leaf surface.

## Introduction

The relationship between plants and microbes is a multifaceted one. Plant–microbe interactions reach our attention most effectively when they are negative—for example, when crop diseases threaten harvests and livelihoods, or when epidemics such as chestnut blight or ash dieback cause unmissable alterations to landscapes and ecosystems [1, 2]. However, microbes are ubiquitous in natural environments, as components of soil [3–7], in water [8, 9] and even as bioaerosols [10, 11]; they are also key mediators of earth system processes such as the carbon, nitrogen and water cycles [11–15]. As a result, plants constantly encounter a diverse array of microbes across all three domains of life, and yet the vast majority of these interactions do not result in disease. Some plant–microbe interactions are symbiotic, and, again, we notice those where the plant benefits from the presence of the microbe. These include the closely co-evolved partnerships between plants and mycorrhizal fungi or nitrogen-fixing bacteria [16–18]. However, plants are populated by a whole microbiome of more loosely associated microbes [19, 20] and are further influenced by the community of microbes inhabiting that portion of the soil close to and affected by the plant roots (the rhizosphere) [21, 22]. These microbes influence the plant in a range of ways. Particularly well-studied examples include the plant growth promoting rhizobacteria (PGPR) and endophytic fungi; both of which are implicated in disease and stress resistance, crop yield, nutrient acquisition, flowering time and even plant species’ ranges [23–25]. Beyond this, research efforts over several decades have revealed many layers of interdependence between plants and their associated microbes, to the extent that the collective term ‘holobiont’ has been applied to the host plant and its microbiome [24, 26–28]. Plants present various microbial niches [29–32], collectively termed the rhizosphere (below ground) and phyllosphere (above ground). The phyllosphere includes the phyllo-endosphere and the phylloplane (surfaces of e.g. leaves, stem, flowers, fruits and seeds) [33]. Each niche hosts microbial mutualists, commensals and pathogens, facilitating a range of microbe-microbe and microbe-microbe-plant interactions [34–36]. Plants influence microbial communities in a species- and genotype-dependent manner, via secondary metabolites, exudates and immune responses, all of which vary with developmental stage, plant compartment and biotic interactions [31, 35, 37–42].

The phyllosphere microbiome has historically been neglected by researchers, who have focused on the soil and rhizosphere microbiomes, but it is now appreciated that the phyllosphere represents the largest terrestrial microbial habitat on Earth [43, 44] and must be studied for full understanding of global microbial ecology and even earth systems, in addition to the more obvious research areas around plant health. The combined surface area of the phyllosphere hosts in the region of 10^26^ bacteria, plus yeasts, filamentous fungi, algae, viruses and protists [45–48]. Further, the phyllosphere represents a hotspot for microbial evolution and genetic exchange [49–52] and is a significant source of microbes in other environments, including soil, water and atmosphere, with roles in the major earth cycles. This review will therefore focus on phyllosphere microbes. Bacterial communities are the best studied in this niche; here, we will consider them alongside fungal communities and discuss the ways in which current knowledge of the factors that shape bacterial communities on leaves, and their relationships with plant host, might be extrapolated to fungal communities, including their relationships with the host and with bacterial communities. We will consider the wheat pathogenic fungus, *Zymoseptoria tritici*, as a case study, discussing the importance of its epiphytic growth phase and potential interactions with phyllosphere microbiota.

## Reaching the leaf surface

Immigration of epiphytic microbes into the phyllosphere is either seed-mediated—known for some fungi, bacteria and viruses [44, 53–55], or environmental—from soil, air movement, or rain-splash, which can transmit microbes from soil and from neighbouring plants [45, 56–58]. Seed-mediated colonisation gives access to host tissues from the outset of the plants’ life, providing scope for long-term co-evolution between microbes and their hosts [44, 59]. Acorns, for example, contain a diverse community of microbes whose spatial distribution facilitates non-random transmission to the rhizo- and phyllospheres, and the seedling phyllosphere closely resembles this acorn microbiome [60]. However, the importance of the seed-borne microbiome falls as the plants mature [55, 60]. A field study which combined carefully controlled plant age and development with microbiome transplant experiments demonstrated that neighbouring plants significantly affect epiphytic bacterial community composition [41]. In other field studies, episodes of heavy rain or strong winds were found to alter the microbial communities on leaves, as did irrigation [46, 61, 62]. Bacteria enter the atmosphere from leaf surfaces and cryptogamic coverings of rocks and soils [10, 11, 48] and are able to survive in the boundary layer and travel widely, even between continents [10, 11], as can spores of some fungi—a factor in outbreaks of the globally emerging, aggressive strain of the wheat rust fungus *Puccinia graminis* (Ug99) [63–65] and of the pan-European spread of the ash dieback fungus, *Hymenoscyphus fraxineus* [1, 64, 66]. Although estimates vary widely and are dependent upon vegetation type and climatic factors, a flux of around 100 bacteria and fungal spores entering the atmosphere every second for every square meter of land is an accepted approximation [10]. In one study, 7% of the bacteria on spinach leaves belonged to the genus *Massilia*, a common airborne bacterium [11, 67]; while this alone is not proof of the route by which these bacteria populated the leaf surface, it supports the idea that airborne inoculum contributes to epiphytic communities. Thus, the leaf surface microbiome will be influenced by many factors, including both local and global microbe pools and weather conditions, as well as host factors like species and genotype.

## Leaf surface community assembly, development and structure

Once on the leaf surface, microbes are exposed to many stressors [45, 68–72]. Leaves are light-harvesting organs, so exposure to ultraviolet radiation is inevitable, although fluxes change rapidly, as do associated environmental factors such as temperature and humidity [36]. In addition, plant defences activation may lead to reactive oxygen (ROS) stress [45, 71, 73]. The leaf surface is also believed to be an oligotrophic environment where access to carbon, nitrogen and micronutrients may be growth-limiting and subject to competition from other resident microbes [74–77], as suggested by an over-representation of genes related to aerobic anoxygenic phototrophy in some epiphytic bacterial communities [76]. Phylloplane microbes must also contend with a multitude of anthropogenic chemical inputs from agricultural practices (fertilisers, pesticides and pollution) [78, 79]. The phylloplane is a dynamic environment [80] and microbes can persist only if they are adapted to its challenges.

Leaf surface microbiomes differ from those in the surrounding soil and rhizosphere of the same plants, suggesting that the leaf surface is a selective environment [48, 49, 71, 73, 81]. Bacterial communities are generally dominated by four phyla—Proteobacteria, Firmicutes, Actinobacteria and Bacteroides [46, 76, 82], a pattern that has been shown to hold true across monocots including wheat, rice and switchgrass [54, 82], annual dicots such as *Arabidopsis*, clover, lettuce and spinach [67, 82–84], perennials including coffee [85] and various tree species [68, 86–88], as well as across temperate, Mediterranean and tropical climate zones [68, 89]. Fungi are often described as transient or ephemeral on leaf surfaces and only present as spores [71, 90], but this overlooks a rich community of largely basidiomycete yeasts, which, like their bacterial neighbours, are generally represented by a subset of possible classes—especially Cystobasidiomycetes, Tremellomycetes, Microbotryomycetes and Uridinomcyetes including *Cryptococcus*, *Sporobolomyces* and *Rhodotorula* [45, 48, 54, 91–95]. Epiphytic yeast populations are estimated to reach around 10^5^ cfu per gram of leaf and are understudied, with recent studies uncovering new species, genera, families and even orders [95–98] and demonstrating significant interactions with other microbes, including plant pathogens [99]. A few classes of largely ascomycete dimorphic and filamentous fungi are also frequent members of epiphytic communities—especially Leotiomycetes, Dothideomycetes and Sordariomycetes [45, 48, 54]. Notable epiphytic genera and species include the bacteria *Pseudomonas*, *Methylobacterium* and *Sphingomonas* [32, 45, 48, 100], the yeast *Aureobasidium pullulans* [45, 90] and filamentous fungi *Acremonium*, *Alternaria*, *Aspergillus*, *Cladosporium*, *Mucor* and *Penicillium* [45, 54, 90].

Leaf surface microbiomes are less diverse than bioaerosols or soil microbiota [11, 101–103]. Hypotheses concerning the factors most important in shaping them include dynamic exchange with other niches, selection for abiotic stress tolerance, or microbe selection by the plant. It is argued that the similarity of microbial communities across a wide range of plants and habitats is an indication that plants actively recruit and maintain their epiphytic microbiota [54]. This idea is supported by evidence that microbial communities depend on host species and genotype [43], and that as leaves age, their microbiome loses diversity, suggesting that specific microbial genotypes are selected on the leaf [38, 104, 105]. Possible mechanisms for recruitment and selection by the plant include chemical profiles of leaf waxes, exudates and volatiles [43, 106, 107] as well as physical properties [43]. Plant immune responses may also play a role, since immune mutants of *Arabidopsis* exhibited altered phyllosphere microbiomes [73]. There is also evidence that plants under stress use a chemical ‘cry for help’ to recruit beneficial microbes [34, 108]. Set against the idea the microbiomes are determined by their hosts, however, is evidence for geographical endemism, particularly in fungal epiphytes [45], and for the importance of neighbouring plants and seasonal succession in shaping leaf surface microbiomes [41, 56, 58, 103, 109, 110], although it should be noted that leaf properties themselves change seasonally and according to plant development and age [37, 111–113].

Guo et al. [42] assessed the impact of host species, host genotype, host niche and the abiotic factor of water stress on the fungal microbiome of wheat (*Triticum aestivum*) and oat (*Avena sativa*). They reported that host niche had the greatest overall impact on community dynamics, but host genotype and water stress have significant effects on the community structure within niches. Using source-tracing analysis, the authors unsurprisingly identified soil as the source of fungal root communities, but were unable to identify the source of over half of leaf fungi; further, less than 10% of leaf epiphytes were found in other host niches [42]. This study therefore supports the concept that the open nature of the phylloplane is an important factor in microbiome assembly [44].

**Fig. 1:**
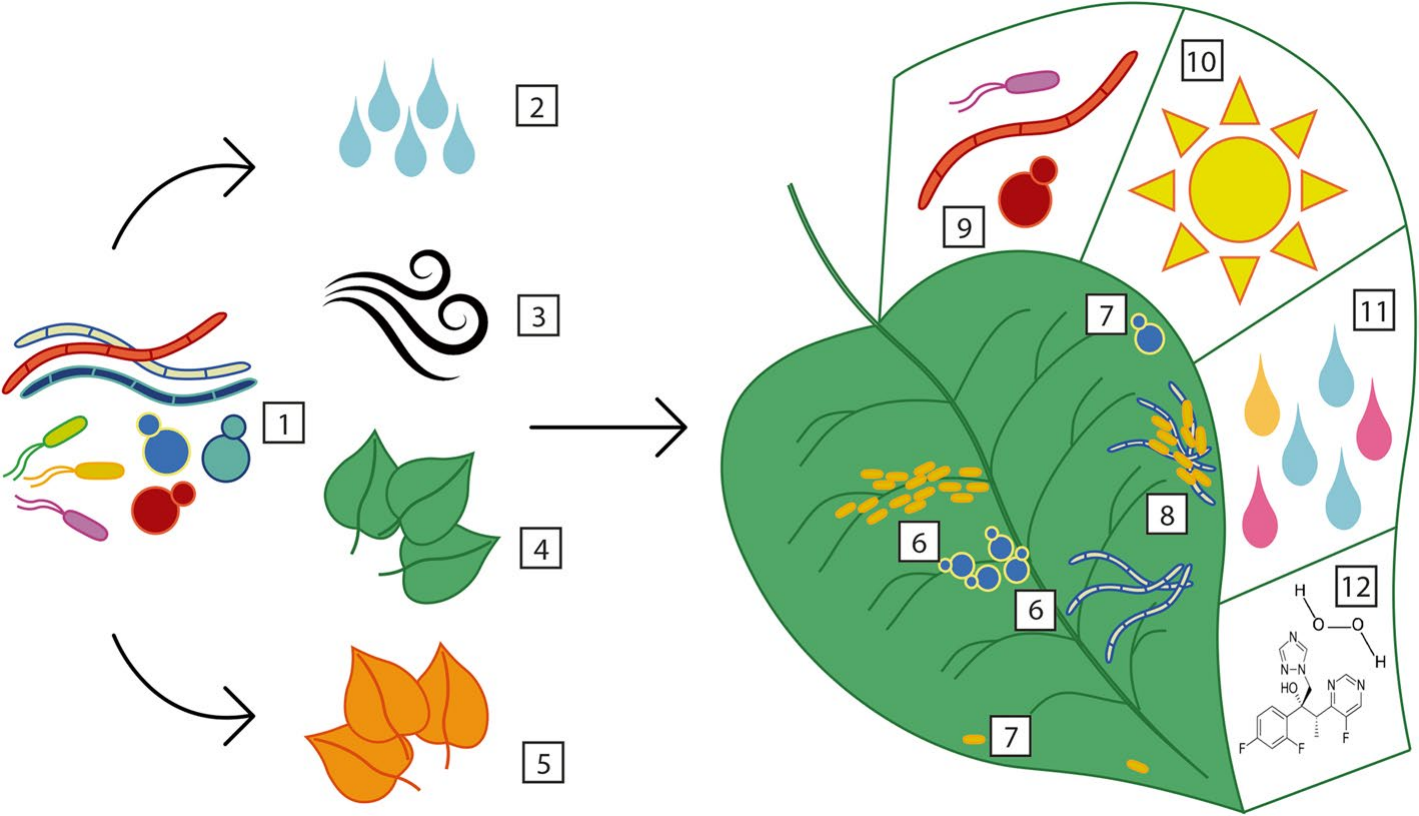
Arrival of microbes on the leaf surface and assembly of epiphytic communities. Diverse microbes (1) land on leaves by a number of different routes, including rain (2) and by air movement (3), as there are large fluxes of microbes into the atmosphere from surfaces including soil, leaves and cryptogamic coverings of, for example, rocks; and many fungal spores and bacteria can survive in the boundary layer and may thus be transported worldwide. Microbes may be deposited directly from the air or rained out of the atmosphere. Rain can also be an additional source of microbes that may be splashed from neighbouring soil and plant surfaces (4), meaning that both the neighbouring plant community composition and seasonal factors (5) can affect the diversity present. Once on the leaf, microbes exist in large aggregates (6) whose location and size are determined by the heterogeneous nature of the leaf surface topology and nutrient distribution, although individuals (7) and microcolonies are also present. Aggregates include mixed species communities which may exist as biofilms (8). Leaves harbour a less diverse microbiome than the surrounding environment, partially due to the effects of stresses such as competition (9), high temperatures, U.V. radiation and drying (10), low and heterogeneous water and nutrient availability (11), and the presence of toxins such as plant defence compounds and agricultural antimicrobials (12). The influence of the host is then proposed to further select a specialised epiphytic microbiome

Xiong et al. [37] made innovative use of artificial leaves to capture the airborne microbes in the local environment of maize plants. This demonstrated an ongoing airborne contribution to phylloplane diversity [37]. However, host developmental stage was found to be the strongest determinant of maize microbiome community composition [37]. Plant developmental stage affects metabolism, leaf exudation, leaf surface physicochemical properties and immune traits, all of which are likely to influence the recruitment and survival of microbes [37, 114–118]. Interestingly, Xiong et al. showed that bacterial soil communities supported plant health and nitrogen uptake in young maize, while fungal roles in soil carbon and phosphorous cycling were seen later in plant development [37]. This could be interpreted as evidence for host-mediated microbiome modulation, a long-proposed mechanism for which evidence has been steadily building [29, 119–123]. It is possible that such mechanisms may also influence leaf surface populations. Factors that shape the leaf surface microbiome are summarised in Fig. 1.

## The leaf surface habitat

The apparently hostile, oligotrophic leaf surface environment is not homogeneous, either physically or chemically. Leaf surfaces are three dimensional and present heterogeneous microhabitats at the microbial scale. This ‘landscape’ is covered by a cuticle that itself comprises crystalline wax structures, which vary with plant species, genotype and development [104]. Leaf macroscopic features such as veins also create an intricate topography [32, 45, 124]. At the microscopic scale, the depressions over the anticlinal cells walls are an important feature which patterns the leaf surface with a network of grooves [32, 45, 125]. There are also additional cell types, including trichomes and the guard cell complexes that surround stomatal apertures. A number of studies have demonstrated that the leaf surface is as heterogeneous in chemical factors, including water and nutrient availability, as it is non-uniform in shape [43, 126–128]. Some of the most elegant of these have used bacterial epiphytes as bioreporters, created detailed topographic leaf surface replicas and/or utilised cutting-edge single-cell imaging techniques, allowing the heterogeneity of the environment to be understood at the scale experienced by the microbial inhabitants [32, 129–132]. Reporters have been used to gain insights into the spatial heterogeneity of sugars, phenolics and water on the leaf surface [124]. Carbon, present heterogeneously on the leaf surface as carbohydrates, amino acids, organic acids and sugar alcohols, is the most limiting factor to epiphytic growth [47]. Simple sugars, thought to leach directly from the leaf interior, are the dominant carbon source on leaf surfaces. These remain detectable in the leaf washings of heavily colonised plants, indicating that not all soluble sugars are accessible to microbes in situ [104, 133, 134]. A sucrose/fructose responsive GFP-based reporter in *Erwinia herbicola* revealed much spatial heterogeneity in these sugars on bean leaves [135], while a fructose utilisation bioreporter based on a short half-life GFP showed that most bacterial cells had exhausted the available fructose within 24 h of inoculation [126]. However, a subset of leaf surface locations supported much longer-term fructose usage, suggesting that those specific areas had much more of the sugar present [126]. A GFP-based biosensor for iron constructed in *Pseudomonas syringae* suggested that bioavailable iron was also heterogeneously distributed, but that many epiphytic bacterial cells did not experience iron limitation [136].

Water availability is also heterogenous and influences the distribution of any dissolved solutes. Free water on the leaf surface is essential for the survival, growth and activity of phyllospheric microorganisms, allowing them to move, communicate and acquire nutrients [70, 137–139]. Water vapour escaping from stomata may create a very thin, laminar water layer over the leaf surface, reducing water stress for epiphytes [104]. The collective body of water on the leaf surface is known as the phytotelma [32, 137]; its size, spread and connectivity are influenced by precipitation, irrigation, condensation, transpiration, guttation and evaporation [32]. Water stress varies across the leaf surface, as water droplets may dry out and shrink, while the structures and topography of the leaf will influence water ingress and retention [32, 140].

Doan et al. [32] used PDMS leaf replicasts to study water distribution on leaves that had been dipped or sprayed. They used a combination of electron microscopy and solute biosensing bacteria to show that water retention is associated with venation and trichomes, and that the diffusion of solutes across a leaf surface preferentially occurs in the direction of the ribs in the leaf created by veins. Bacteria survived better on surfaces whose topography allowed greater water retention, and their physical clustering in the grooves along veins and over anticlinal cell walls, as well as at the base of trichomes, might be explicable solely through the physical effect of these structures on water distribution [32]. A water bioreporter constructed in three bacterial species (*Escherichia coli*, *Pantoea agglomerans* and *Pseudomonas syringae*) showed water stress began to affect cells within 5 min of inoculation onto bean leaves, but also that cells did not experience water stress as extreme as predicted by published psychrometer readings of leaf water potential, suggesting the existence of microhabitats protected from drying [140]. Bacterial cells also respond to water stress, often showing cross-protection with other stressors [141] or the adoption of the resilient ‘persistor’ metabolic state [142].

The chemistry and structure of the cuticle is also an important factor in determining water distribution on the leaf surface. Cuticle permeability is important in epiphyte survival and growth, since it provides water and solutes to the leaf surface [124]. Cuticles are composed of cutin—a polymer of cross-linked hydroxyl fatty acids—and waxes; they are not impermeable to water, as single molecules of water may interact with polar components to pass through the cuticle matrix [143]. While permeation is slow and does not carry solutes across the cuticle, aqueous pores, formed from clusters of polar molecules, exist within plant cuticles and are important for the exudation of nutrients from inside the leaf [48, 124, 143]. Uptake and accumulation of the dye berberine sulphite has been used to visualise the movement of water through plant cuticles [143]. The dye was detected first at the cuticular ledges of stomatal guard cells, and then appeared at anticlinal cell walls, trichomes and over veins [143]. Notably, these areas of higher permeability are also the sites on leaves at which highest microbial densities are found [70, 124, 143].

## Microbial adaptation to the leaf surface

Epiphytes must either tolerate the abiotic stresses associated with the leaf surface or mitigate them through their own actions. For this reason, successful epiphytic microbes must adopt a range of strategies to find or create microhabitats that are protected from abiotic stress [138], including manipulation of their host to modify their environment [144]. The ability to navigate to, and remain in, those niches within the leaf surface that are richest in nutrients and available water is key to epiphytic survival of both bacteria and fungi. Bacterial cells are motile and can move towards favourable locations by chemotaxis following detection of nutrients or signals [42, 68, 90, 145, 146]. Motility is also necessary for virulence in bacterial phytopathogens which invade from the surface, such as *Ralstonia*, *Dickeya* and *Xanthomonas* [48, 90, 147]. Fungal spores are non-motile, but hyphae navigate the leaf surface using polar growth [148–150] which may be directed chemotactically or thigmotrophically [151, 152], allowing them to seek out nutrients and respond to the leaf topography [153, 154]. Movement to the most sheltered and nutrient-dense areas of the leaf where survival and reproduction are highest causes aggregation, with most of the microbes on a leaf surface occurring in aggregates of 1000 cells [47, 155]. Solitary bacteria do occur on leaves in large numbers, but the sheer size of some aggregates means that they may represent as much as 80% of the leaf surface bacteria [104]. These aggregates are often mixed species, including both bacteria and fungi [104, 156, 157].

To survive the abiotic stresses on the leaf surface, many microbes form biofilms. A biofilm may be defined as an aggregation of cells, attached to a surface and embedded in an extra-cellular matrix (ECM) [158–166]. In bacteria, the ECM is usually composed of extracellular polysaccharides (EPS) [54, 90, 167, 168]. Biofilms are resistant to desiccation [103, 145, 169, 170], antimicrobials [104, 171–175] and reactive oxygen species, which are important plant defences [176, 177]. Biofilms can also be formed by yeasts and filamentous fungi [178–183], including a number of plant pathogens [177, 184–186]. Generally, the mechanisms of stress resistance in biofilms are not fully elucidated, but a number of factors are known to contribute, including the expression of efflux pumps for toxins/antimicrobials; the action of the ECM in limiting diffusion of stressor chemicals towards cells and in limiting water loss; the presence of persistor cells within biofilms, and, more broadly, metabolic and transcriptomic heterogeneity among the cells within biofilms [177, 187–192]. For fungal biofilms increased resistance to a number of stresses, such as antifungals, ROS and UV, is documented, and mechanisms proposed are similar to those seen in bacterial biofilms, with ECM components such as beta-glucans and eDNA thought to contribute along with cell–cell heterogeneity in metabolic state, induced by differential resource availability across the biofilm [173, 192–196]. Multi-species and even cross-kingdom biofilms, in which the ECM is derived from both fungal and bacterial secretions, are known and likely the norm on the leaf surface [90, 197–201]. As with single species biofilms, stress resistance is enhanced by a range of mechanisms in these mixed biofilms. The physical proximity of various microbial species in a stressful environment underpins a high rate of horizontal gene transfer (HGT), for which the phyllosphere is a known hotspot, particularly for plasmid and gene cassette exchange, although the mechanisms underpinning increased HGT in the phyllosphere are not completely understood at present [202–208].

Many epiphytic bacteria produce plant hormones such as auxins, gibberellins and cytokinins in order to communicate with and manipulate their host [45, 54]. *Pantoea agglomerans* and *Pseudomonas syringae*, for example, produce the auxin indole acetic acid (IAA) when growing epiphytically [209]. Exogenous auxin application causes the loosening of plant cell walls and release of sugars [210, 211]; it is therefore believed that in planta IAA production is likely to increase the rate of sugar exudation from the leaf [104, 212]. This strategy for accessing carbon can provide a selective advantage—IAA producing *P. agglomerans* grew faster and reached larger populations than a mutant lacking this ability [212]. Production of the plant hormones abscisic acid (ABA) and ethylene can also affect stomatal opening, increasing water availability [45, 213], while cytokinins may trigger the release of methanol, which is metabolised by some bacterial epiphytes [90, 104]. The manipulation of phytohormones is also known in fungal endophytes, although this is often associated with induction of hormone production in the plant [214]. More recently, the direct production of phytohormones by endophytic and epiphytic yeasts and filamentous fungi has been observed [215, 216]. Examples include the production of the auxin indole acetic acid and the cytokinin zeatin by epiphytic basidiomycete and ascomycete yeasts [216, 217]. Phytohormone production is also known in endophytic yeasts, where it has been linked to plant growth promotion and pathogen suppression, although the mechanisms involved remain unclear [215, 216, 218].

To increase nutrient exudation and availability on the leaf surface, some epiphytes produce compounds known as surfactants that increase the wettability of the leaf surface [43, 44, 74, 90, 219–221]. *Pseudomonas syringae* isolates produce syringafactin and syringomycin; syringafactin is hygroscopic and sorbs to leaf cuticle waxes, increasing their permeability to nutrients as well as their wettability [222], while the potent biosurfactant syringomycin induces the formation of host membrane ion channels to induce a flux of metabolites from the cell [223]. While syringomycin is phytotoxic at high concentrations, sub-toxic concentrations are produced by non-pathogenic *Pseudomonas syringae* isolates, indicating a role in epiphytic fitness [224]. Surfactant production is also known in yeasts and fungi, but has been studied to a much lesser extent in these organisms [225–227]. For example, surfactants produced by some *Trichoderma* sp. are thought to have a role in biocontrol activity towards other fungi [226].

Another strategy is the secretion of enzymes such as cutinases, esterases and lipases which can liberate nutrients from the cuticle and, by degrading cuticular components, increase its permeability to nutrients and the plants’ susceptibility to infection [228, 229]. Phyllosphere microbe metaproteomes also show enrichment for ABC transporters, porins and TonB-dependent transport systems, suggesting an enhanced capacity for nutrient uptake [47, 68, 82, 84]. Proteins of the OmpA porin family from gram-negative bacteria are among the most abundant found on the leaf surface [84, 230]. It is also common for epiphytic microbes to produce iron-chelating siderophores to maximise iron uptake; well-known examples include pyochelins, pyoverdines and pseudobactins from *Pseudomonas* species [54, 231]. Siderophore production is also common among epiphytic yeasts, where it is associated with biocontrol activities against fungal pathogens [99, 232].

## Commonalities between epiphytic and phytopathogenic adaptations

Consideration of this suite of epiphytic adaptations raises three important points: firstly, many adaptations to life as an epiphyte often show similarities to the adaptations of biotrophic pathogens, with overlap between epiphytic and biotrophic adaptations such as production of plant hormones, production of surfactants and plant defence suppression; these similarities will be explored further below. Secondly, many of the adaptations necessary for life as an epiphyte moderate the environment around the microbe and can thus be considered public goods; and thirdly, most of these adaptations seem specific to, or have largely been studied in, bacteria and yeasts. Together, these observations raise some interesting possibilities about epiphytic microbial ecology and evolution.

Rather than a binary differentiation between harmless residents and phytopathogens, these traits can be seen as a continuum: many hemibiotrophic bacterial pathogens, in fact, behave as epiphytes during early colonisation of leaves [87, 233, 234]. Virulent *Pseudomonas syringae* plant pathogens, for example, are often differentiated from non-pathogenic epiphytic strains by a single host-specific virulence factor. Strikingly, many of the genes encoding virulence factors, particularly those for effector proteins, are found on plasmids or in pathogenicity islands; the difference between pathogenic and commensal microbe can therefore be the gain or loss of a plasmid [104, 235–237]. There are similar examples of virulence factors being encoded on dispensable or even exchangeable parts of the genome in plant associated fungi, including the transfer of ToxA from *Parastagonospora nodorum* to *Pyrenophora tritici-repentis* [238].

In *Pseudomonas syringae*, as in other plant-associated bacteria, many traits that are key to both epiphytic and pathogenic lifestyles are under quorum sensing (QS) regulation [145, 219, 231]. These include swarming motility, EPS secretion, siderophore secretion, production of the phytotoxin coronatine and delivery of effectors via the type three secretion system (T3SS) to disarm host immunity [145, 219, 231]. QS is a process in which bacteria produce and respond to specific molecules whose concentration in the environment is a proxy for population density [48, 145, 219]. On the leaf surface, QS regulation of these virulence determinants plays a major role, not only in determining whether bacteria will proliferate epiphytically or become virulent, but also in co-ordinating these processes with abiotic conditions. On the heterogeneous leaf surface, water is unevenly distributed [32, 140]. The concentration of QS signals thus depends, not only on microbial numbers, but also on the volume of water. QS signals repress motility in *Pseudomonas syringae* [219], an epiphytic adaptation which helps the bacteria to remain in pockets of available water and nutrients, where their populations can expand [145, 219]. Quorum sensing is also known in many fungal species, where it can be important in determining growth form [239, 240]. Interestingly, fungal QS signals, such as farnesol, are also involved in biofilm formation in some fungi, and both fungal and bacterial QS signals were recently shown to induce biofilm formation by *Ophiostoma piceae*, including in mixed-kingdom biofilms formed in consortium with the bacterium *Pseudomonas putida*. Given the importance of biofilm formation, including cross-kingdom biofilm formation, on the leaf surface, this suggests that QS molecules from both bacteria and fungi may play an important role in determining colonisation success and stress survival in epiphytic microbial communities. Further, volatile QS signals have been identified from *Fusarium culmorum* and *Cochliobolus sativus* and may show promise in retarding phytopathogen growth [241].

## Interactions between epiphytic microbes

A large proportion of leaf surface microbes occur in the same conducive microhabitats [83, 104, 126] and may collaborate intimately in multispecies biofilm formation [90, 197–199]. Microbe-microbe interactions in the phyllosphere are not always collaborative—under resource limitation they are, of course, often competitive, and another epiphytic adaptation is the production of antimicrobials [34, 71, 108, 231, 242–245]. These interactions, and their potential exploitation for biocontrol of phytopathogens, are reviewed in detail elsewhere [243, 246, 247]. Whatever their relationship, the presence of other epiphytic microbes will change the environment experienced by each. For instance, increased nutrient exudation and diffusion due to surfactants make nutrients available to all microbes in the vicinity while siderophores can be taken up by any organism with the correct receptor. This has clear implications for microbial interactions on the leaf surface. In the next section of this review, we consider the possible interactions between the epiphytic microbiome and an economically important plant pathogen, the fungus *Zymoseptoria tritici*, which spends considerable time as an epiphyte and so provides a particularly interesting case study. We propose that the research effort that has been put forward in understanding this crop pathogen could be leveraged by using *Z. tritici* as a model system in studying microbe-microbe and microbe-microbe-plant interactions on the leaf surface.

## *Zymoseptoria tritici* as a case study and potential model system for epiphytic fungi-bacteria interactions

Filamentous fungi are often considered ephemeral members of epiphytic communities, largely because their success in planta is often predicated on rapid entry into the leaf, either through stomata or by direct penetration [248–250], and it is generally assumed that once the spores of these fungi germinate, there is a short window in which the fungus must enter the leaf interior or starve [251–253], leading to adaptations for rapid leaf entry. *Puccinia graminis*, for example, follows the anticlinal cell walls of leaves until a stomatal entry point is found, while *Magnaporthe oryzae* ignores stomata, instead entering the leaf using a high-pressure injection system—the appressorium [254, 255]. The perception that such adaptations are universal in plant-associated fungi is likely a product of the intensive research effort into a relatively small number of economically important plant pathogenic fungi for which this is true [256]. However, there are filamentous fungi that are resident on the leaf surface for part or all of their lifecycle, and do not fit the paradigm of ‘sense host, germinate, penetrate’ at all. Examples include the sooty blotch and flyspeck fungi that grow on apples post-harvest [257] and biofilm forming growth of various fusaria [184–186, 196]. It is not currently known whether these filamentous fungal epiphytes possess similar adaptations to those seen in bacteria, as these fungi are neither economically devastating pathogens nor known as candidates for biocontrol of such pathogens, meaning that they have received little research attention.

**Fig. 2:**
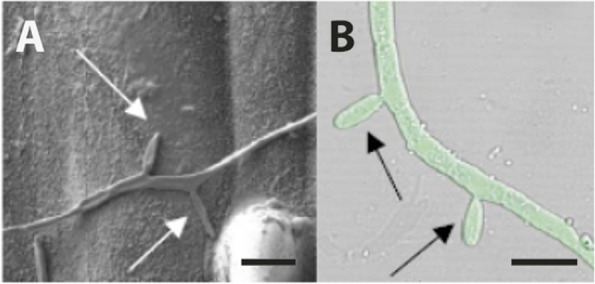
Microcycle conidiation in *Zymoseptoria tritici*. Budding can occur from blastospores in a variety of environments. **A** Cryo-scanning electron micrograph of a blastospore of *Zymoseptoria tritici* IPO323 5 days after inoculation onto a wheat leaf. While the spore has germinated to form a hypha (left), there are also two buds forming (white arrows), which represent the production of new blastospores. **B** Epifluorescence micrograph of a *Zymoseptoria tritici* IPO323 blastospore carrying a cytoplasmic GFP construct, 3 days after inoculation into minimal medium on a glass slide. Again, buds are forming directly form the blastospore (black arrows). Scale bars represent 10 mM

**Fig. 3:**
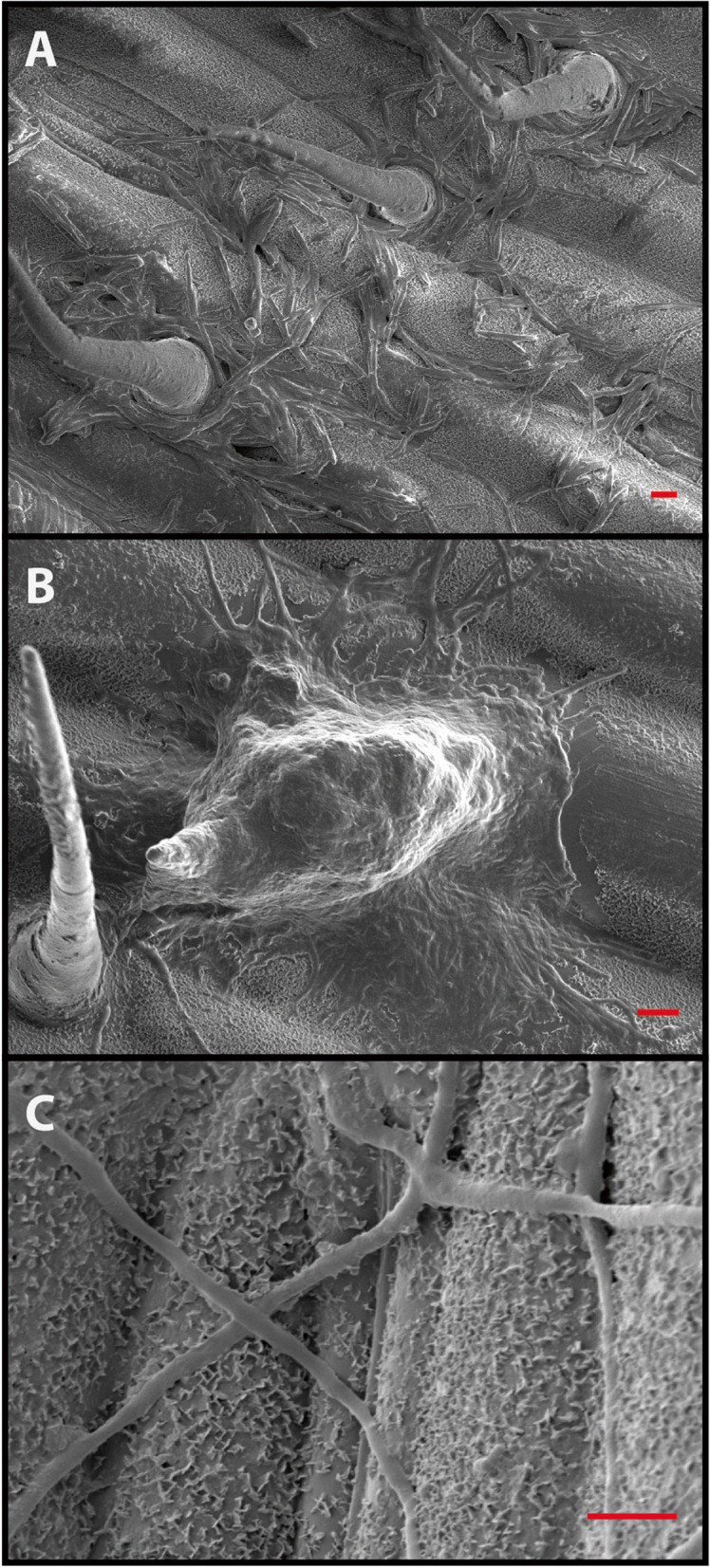
Leaf surface topology affects fungal colonisation. Scanning electron micrographs showing the effect of leaf topology and surfaces on the epiphytic growth of the phytopathogenic fungus, *Zymoseptoria tritici* IPO323. **A** Fungal proliferation on the leaf surface is spatially heterogeneous. Spores are ‘captured’ by trichomes and proliferate by both hyphal and budding growth into aggregates around the base of these specialised cells, possibly due to physicochemical differences such as water availability or the presence of leaf exudates in this microhabitat. **B** *Z. tritici* can form biofilms on leaf surfaces; here, a mass of cells embedded in ECM can be seen surrounding a wheat trichome. **C** Hyphal growth in *Z. tritici* is not obviously directed by leaf features; here, one hypha follows and anticlinal cell wall while others are not oriented to topological features. Hyphae have crossed a stomatal aperture without penetrating the leaf. Scale bars (red) = 5 mM

A recent development may, however, mean that epiphytic adaptation has become relevant for a full understanding of the lifecycle of an important crop pathogen, *Zymoseptoria tritici*, the causal agent of *Septoria tritici* leaf blotch (STB) of wheat [258]. Until recently, this fungus was believed to behave like other plant pathogenic fungi, germinating on wheat leaves and finding its way into the leaf through the stomata within 24–72 h [259–264]. However, no adaptations for rapid entry comparable to those in other plant pathogenic fungi have been found in *Z. tritici*. Most studies have reported random or untargeted growth [260, 265, 266], with hyphae often growing over stomata if they are closed (Fig. 3). In 2017, a study by Fones et al. [265] indicated that virulent isolates could spend up to 10 days on the leaf surface under optimal conditions, during which time hyphae grew over the leaf surface at random before entering through stomata or wounds [265, 267]. This was corroborated by studies that showed asynchronous germination on the leaf surface, followed by a variable but often prolonged period of epiphytic growth [268], which, in fungal isolates of equal virulence, varied in extent and morphology [269]. Blastosporulation (microcycle conidiation; Fig. 2) and anastomosis were shown to occur on the leaf surface [270] and epiphytic proliferation was described in isolates growing on resistant wheat that they could not penetrate [271]. Microcycle conidiation represents the simplification of a fungal life cycle in which the fungus produces spores directly from spores, rather than from mycelia [272]. This provides a way of building the population when spore germination and hyphal growth are not favourable, such as on a resistant host [271]. Avirulent isolates were also shown to contribute to sexual reproduction in planta even without penetrating leaves [273, 274]. Together, these findings indicate that *Zymoseptoria tritici* has an epiphytic stage in its lifecycle, for which it is likely to possess adaptations. Thus, this fungus, which has received significant research effort [260, 275–282] and for which molecular and genetic tools are available [278, 279, 283, 284], can be considered a candidate model system for the interaction of a filamentous fungus with the leaf surface microbiota. It is known that *Z. tritici* manipulates the wheat defences to create ‘systemic induced susceptibility’ once it has established infection, and that this involves the induction of dysbiosis [285], but so far little or nothing is known about its interaction with epiphytic microbes.

## *Zymoseptoria tritici:* epiphytic adaptations and potential interactions with the phylloplane microbiome

Many of the adaptations seen in epiphytic bacteria and yeasts have been detected or can be hypothesised in *Z. tritici*. For instance, pycnidiospores of *Z. tritici* have been shown to produce cutinases on the leaf surface. These enzymes are thought to play a role in initial adhesion to the leaf [259, 264, 286], but their action is likely to increase cuticle permeability and thus nutrient exudation, as with bacterial cutinases and esterases [228, 229]. Transcriptome analysis has revealed other *Z. tritici* enzymes potentially involved in acquiring nutrients during epiphytic growth, including peptidases, pectinases, lipases, cellulases, hemicellulases and xylanases [259, 264, 286, 287]. Some of these enzymes may be linked to scavenging freely available nutrients; however, they may also be involved in the acquisition of nutrients via physical breakdown of the plant itself. Transcriptomics has also revealed the upregulation of genes responsible for the secretion of four hydrophobins, proteins involved in the interactions between hyphae and hydrophobic surfaces—these may be involved in leaf attachment [288]. Leaf architecture may also play a part in adhesion (see Fig. 3): leaf washing led to the enrichment of trichome-associated *Z. tritici* [265], and preferential blastospore location in stomatal depressions and around trichomes was also observed [289].

**Fig. 4:**
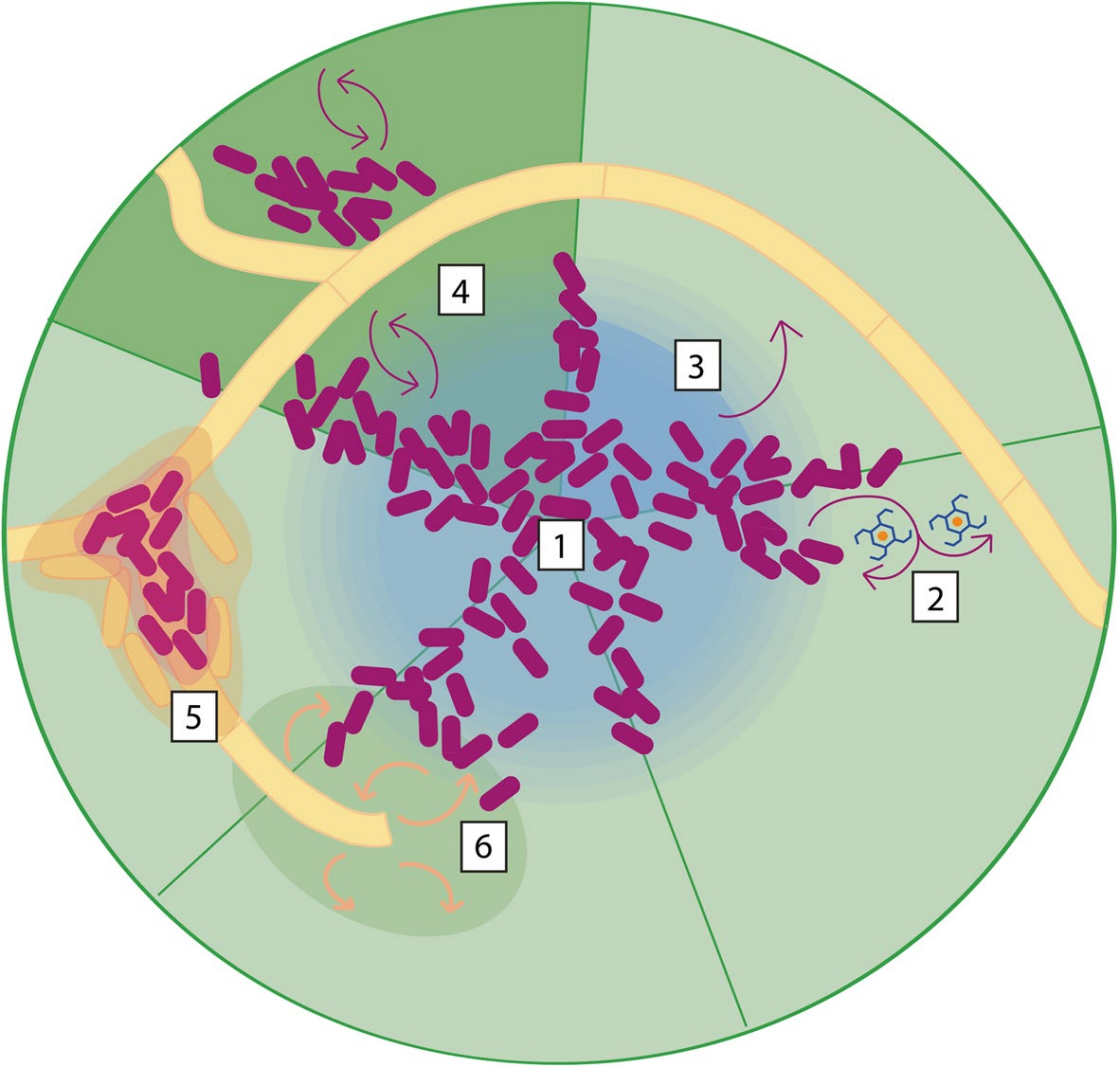
Plant–microbe and putative microbe-microbe interactions on the leaf surface. Shown is a stylised, simplified leaf surface with resident fungal (e.g. *Zymoseptoria*) hyphae and bacterial (e.g. *Pseudomonas*) microcolonies. Leaf surfaces are heterogeneous and contain diverse microhabitats. Areas such as those over anticlinal cell walls where neighbouring cells meet, for example, often have higher nutrient and water availability compared to the leaf as a whole and support greater numbers of microbes (1). Epiphytic microbes have many adaptations to life on the leaf, including secretion of cuticle and cell wall degrading enzymes, expression of transporters, production of plant hormones and biosurfactants and formation of stress-resistant biofilms. In low iron conditions, for example, many microbes produce siderophores, which bind iron with high affinity; the complex is then taken up by either the original producer or other nearby microbes (2). Many bacteria (purple) produce surfactants which increase the wettability of the cuticle (3), increasing nutrient exudation from the plant and increasing nutrient diffusion across the surface. Others produce plant hormones such as the auxin indole acetic acid (IAA) which loosen plant cell walls and increase nutrient efflux onto the leaf surface (4). Increases in nutrient exudation (4) and exudate diffusion (3) benefit nearby microbes, as well as those responsible for the effect. Both bacteria and fungi can secrete and become embedded in a protective extracellular matrix, forming biofilms (5) which may be single species, mixed, or even cross-kingdom. Many fungi also secrete cell wall or cuticle degrading enzymes (6) which, like auxins, increase the permeability of the leaf and the rate of nutrient exudation, as well as directly liberating metabolites. This may attract motile, chemotactic microbes and may play a role in recruiting bacteria to cross-kingdom biofilms (5)

It is notable that these zones of increased adhesion or multiplication by *Z. tritici* coincide with the leaf areas identified as richer in water and nutrients, and consequently hosting bacterial and yeast aggregates in relatively high densities. However, unlike bacterial epiphytes, it remains to be determined whether *Z. tritici* obtains nutrients from its host prior to penetration. A carbon source repressed GFP construct showed bright fluorescence on the leaf surface, hence no evidence of carbon uptake during epiphytic growth [289]. Rudd et al. also failed to find evidence for hexose or nitrogen assimilation during the first 8 days of leaf contact in transcriptomic and metabolomic studies [264]. These findings contrast with the transcriptomic evidence for expression of leaf surface degrading enzymes described above. Interpretation of population-level transcriptomes is now known to be complicated in *Z. tritici* due to the asynchronous behaviour of the fungus in planta [265, 268, 269], but the results from the carbon utilisation bioreporter appear convincing, and it is plausible that *Z. tritici* does not rely on the leaf to provide carbon. A recent study showed that *Z. tritici* remains viable and virulent after 49 days suspended in pure water or in soil [290]. This work demonstrated that *Z. tritici* relies on stored lipids during this extended period of starvation. Thus, carbon uptake on the leaf surface may not be necessary. In that case, it is possible that the action of cutinases, esterases and other cuticle-modifying enzymes, increasing the permeability of the cuticle and with it, nutrient exudation from the leaf, has another purpose. One obvious possibility is that *Z. tritici* does take up and rely upon host-derived nitrogen, which cannot be supplied by lipids. Alternatively, cuticle modification may act to recruit other microbes to areas of *Z. tritici* leaf surface colonisation. It is tempting to speculate that recruitment of bacteria able to increase the wettability of the leaf might facilitate hyphal growth across the surface or that *Z. tritici* might benefit from bacterial siderophore production. *Z. tritici* germinates and grows on many substrates [275], including non-host leaves [291], suggesting that it lacks the host perception abilities of other fungal phytopathogens. However, the slow rate of hyphal extension on non-host tobacco leaves, when compared to wheat leaves, paradoxically indicates some host-specificity [291]. Again, it is tempting to speculate that this difference might reflect different host microbiota, if the fungus is not relying on the host directly. Another conceivable interaction between *Z. tritici* and other epiphytic microbes is the development of mixed species or cross-kingdom biofilms in which multiple epiphytes contribute to the production of the protective ECM (Fig. 4). Biofilms were recently discovered in *Z. tritici* in vitro [177], and large aggregations of *Z. tritici* cells were described in planta for both ‘necrosis-inducing with reduced pycnidiation’ (‘NIRP’) isolates [271], and to a lesser extent, for some virulent isolates on susceptible wheat [269]. *Z. tritici* is generally thought to be highly host specific, causing infection only on wheat and only on specific cultivars. However, thorough review of the literature revealed that the fungus has in fact been isolated from another 26 different grasses, with 6 being probable secondary hosts [292]. Recruitment of epiphytic ‘collaborators’ might protect *Z. tritici* from host immunity or abiotic stress in hosts to which it is less well-adapted.

However, not all microbes encountered will be beneficial. *Z. tritici* produces chloroperoxidases in planta, which may be involved in the production of antibiotics used to control competitive microbes in the phyllosphere [264, 293, 294]. There is also evidence of defence against competitive microbes via antibiotic detoxification—a study by Levy et al. [295] found that three catalase isozymes and one superoxide dismutase are synthesised by the fungus in response to 1-hyroxyphenazine, an antibiotic secreted by *Pseudomonas aeruginosa*. It seems likely that full understanding of the epiphytic phase of *Z. tritici* will require better insights into the relationships between this plant pathogen and other phyllosphere inhabitants (Fig. 4).

## Conclusions and future directions

It is clear that the leaf surface is an important microbial habitat that has, until fairly recently, been overlooked by researchers interested in plant health and the control of phytopathogens. It is now understood that the microbial communities there are crafted by the host plant, but also influenced by and connected with much broader factors, such as neighbouring plants, soil, water and air, with influence even at the level of the earth’s systems. However, at the scale experienced by individual microbes, the leaf surface is an intricate landscape with variable and topographically dictated availability of water, nutrients, mutualists and competitors. Microbes show exquisite suites of adaptations that manipulate the host plant and improve access to nutrients. These are best understood in bacteria such as *Pseudomonas syringae*, but intriguing findings about the phenotype and transcriptome of the fungal plant pathogen *Zymoseptoria tritici* suggest that some similar adaptations may be present, for example for increasing cuticle permeability and breaking down cuticle components. The evidence suggests that *Z. tritici*, however, does not necessarily obtain or rely on carbon from the host plant, raising the possibility that carbon liberated from the host cuticle could be used to recruit chemotactic, mobile bacteria, with which the fungus may then interact. This would make *Z. tritici* an excellent model system for research into microbe-microbe-plant interactions on the phylloplane, as well as opening up potential avenues for biocontrol of this economically important plant pathogen.

## Data Availability

N/A.
